# Biomechanical comparison of bi- and tricortical k-wire fixation in tension band wiring osteosynthesis

**DOI:** 10.1186/s40001-019-0392-7

**Published:** 2019-10-08

**Authors:** J. Nowotny, F. Bischoff, T. Ahlfeld, J. Goronzy, E. Tille, U. Nimtschke, A. Biewener

**Affiliations:** 1 Orthopaedic-Traumatology Centre (OUC), Carl Gustav Carus University Dresden, Technical University, Fetscherstraße 74, 01307 Dresden, Germany; 2 Centre for Translational Bone, Joint and Soft Tissue Research, Dresden, Germany; 3 Institute of Anatomy, Carl Gustav Carus University, Technical University Dresden, Dresden, Germany

**Keywords:** Elbow, Tension band wiring, Olecranon, Fracture, k-wire fixation, Biomechanical study

## Abstract

**Background:**

Patients with a simple transversal fracture of the olecranon are often treated with a tension band wiring (TBW), because it is known as a biomechanically appropriate and cost-effective procedure. Nevertheless, the technique is in detail more challenging than thought, resulting in a considerable high rate of implant-related complications like k-wire loosening and soft tissue irritation. In the literature, a distinction is generally only made between transcortical (bi-) and intramedullary (mono-) fixation of the wires. There is the additional possibility to fix the proximal bent end of k-wire in the cortex of the bone and thus create a tricortical fixation. The present study investigates the effectiveness of bi- and tricortical k-wire fixation in a biomechanical approach.

**Methods:**

TBW of the olecranon was performed at 10 cadaver ulnas from six donors in a usual manner and divided into two groups: In group 1, the k-wire was inserted by bicortical fixation (BC), and in group 2, a tricortical fixation (TC) was chosen. Failure behavior and maximum pullout strength were assessed and evaluated by using a Zwick machine. The statistical evaluation was descriptive and with a paired *t* test for the evaluation of significances between the two techniques.

**Results:**

The average age of the used donors was 81.5 ± 11.5 (62–92) years. Three donors were female, and three were male. Ten k-wires were examined in BC group and 10 in the TC group. The mean bone density of the used proximal ulnas was on average 579 ± 186 (336–899) HU. The maximum pullout strength was 263 ± 106 (125–429) N in the BC group and increased significantly in the TC group to 325 ± 102 (144–466) *N* [*p* = .005].

**Conclusion:**

This study confirms for the first time biomechanical superiority of tricortical k-wire fixation in the olecranon when using a TBW and may justify the clinical use of this method.

## Background

Fractures of the olecranon are with 7–10% of upper extremity injuries a frequent injury in adults [5, 6]. In general, fractures with a dislocation more than 2 mm should be treated with surgery. In the literature, different operative fixation procedures after open reduction of an isolated olecranon fracture are described. Possible methods are tension band wiring (TBW), angular stable plating, transcutaneous screw fixation, intramedullary nailing procedures, and anchor/suture fixation of avulsion fractures [1]. However, simple transversal fractures without dislocated joint component are often treated with an open reduction and internal fixation (ORIF) by using a TBW, which was first described by Weber and Vasey [13]. In general, the principle of the TBW means, that under axial load by muscle tension always compressive and tensile forces arise on the involved bone. In the case of a fractured bone, this always leads to a gap of the fracture on the traction side. The tensile forces can be neutralized with a TBW and converted into compressive forces. This technique ensures a dynamic compression of the fragments and a rapid healing of the bone. TBW is a standardized surgical technique, which is easy to learn and can be performed with little instrumental effort. At the same time it has shown excellent functional results [3]. The major disadvantages of these techniques are postoperative irritations caused by the implanted hardware and secondary dislocations of the k-wires [1, 4, 10, 14]. This can cause consecutive irritation or perforation of the skin and in the end a soft tissue infection [3]. Painful prominences of the k-wires are one of the most frequent postoperative complications and result in about 80% an implant removal.

However, the high rate of irritation emphasizes the importance of correct use of the TBW, because the secondary dislocation is often caused by an incorrect or insufficient application of the wires. Thus, it is assumed that a loosening of the wire may cause a sliding of the fracture on the wire and occur a secondary dislocation. It should be therefore ensured that the wires are securely fixed in the bone and do not allow any mobility. Care must be taken to ensure that the wires are bent at their ends and fixed securely in the cortex; otherwise, the construct can fail.

**Fig. 1:**
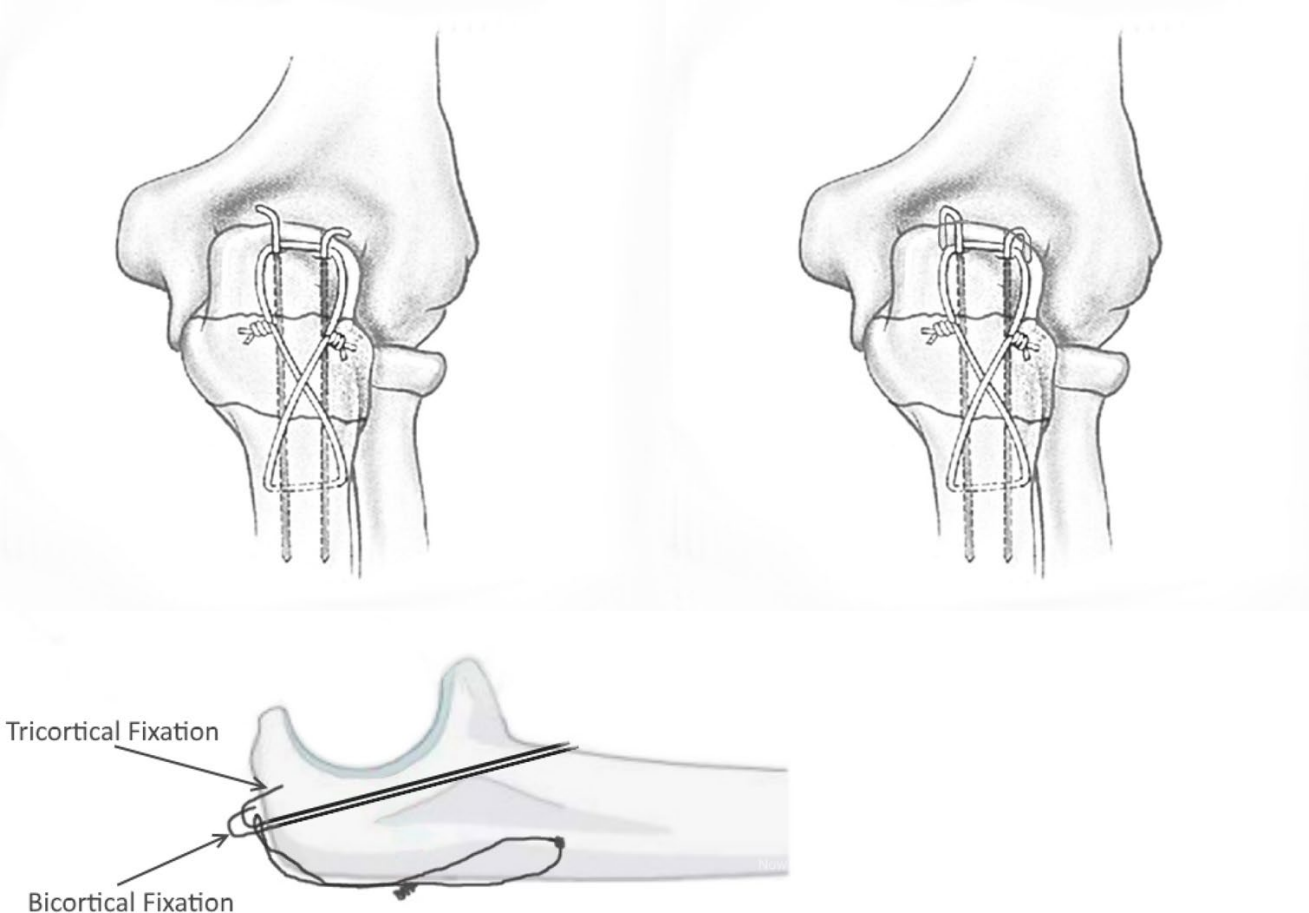
Schematically drawing of the bi- (**a**) and tricortical fixation (**b**) of the k-wires while TBW and how it was used in the present study (**c**)

**Fig. 2:**
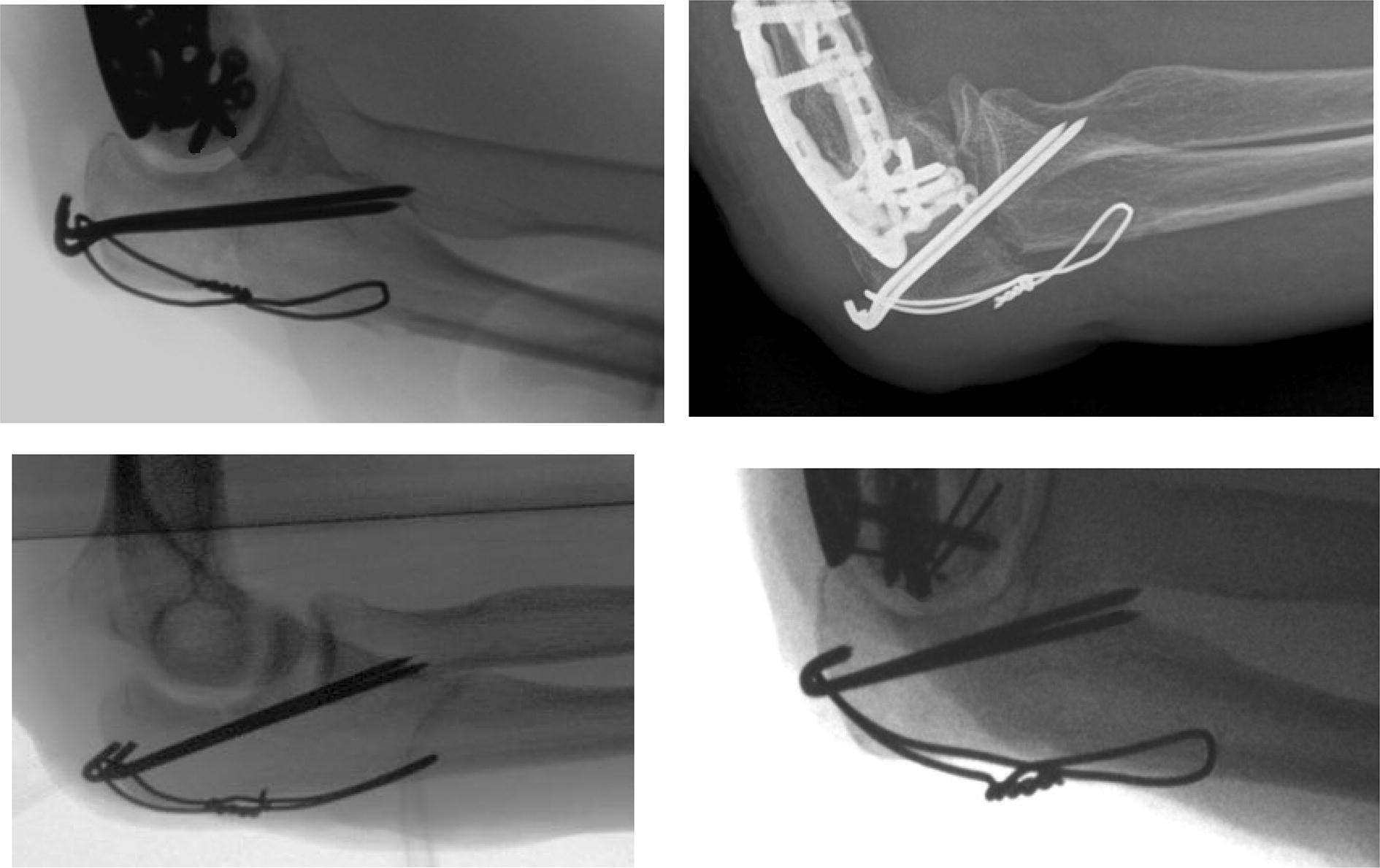
Clinical example of failure after bicortical fixation (top row) and regular X-ray after tricortical fixation (bottom row)

In the literature, a distinction is generally only made between transcortical (bi-) and intramedullary (mono-) fixation of the wires. Saeed et al. already showed the significant advantage of transcortical versus intramedullary fixation in regard to secondary dislocation of the k-wires in a clinical/radiological study [8]. There is the additional possibility to fix the proximal bent end of k-wire in the cortex of the bone and thus create a tricortical fixation (Figs. 1 and 2). In the current literature, there is usually no differentiation between bi- and tricortical fixation of k-wires and generally is spoken of transcortical fixation. In the case of tricortical fixation, the perfectly 180° bent k-wire is fixed into the bone at the olecranon tip by pushing with a rush pin impactor. This increases, on the one hand, the stability of the wire itself in regard to secondary dislocation, and on the other hand, it avoids a gliding of the fracture fragment on the wire that leads an osteosynthesis failure (Fig. 2). However, there is no study that could objectify and substantiate this clinical and radiological fact in a biomechanical study.

The aim of the present study is therefore the investigation of the biomechanical pullout strength of the k-wire fixation while using TBW to answer the question, whether a tricortical fixation is more stable than a bicortical fixation. The hypothesis to be investigated is: “The tricortical is compared to the bicortical fixation biomechanical more stable in the use of TBW”.

## Methods

### Specimens

Ten fresh-frozen ulnae were provided by the Institute of Anatomy of University Clinic, Technical University Dresden, Germany. The specimens were frozen at − 22 °C after removal (Liebherr Typ 40073 1, Germany).

**Fig. 3:**
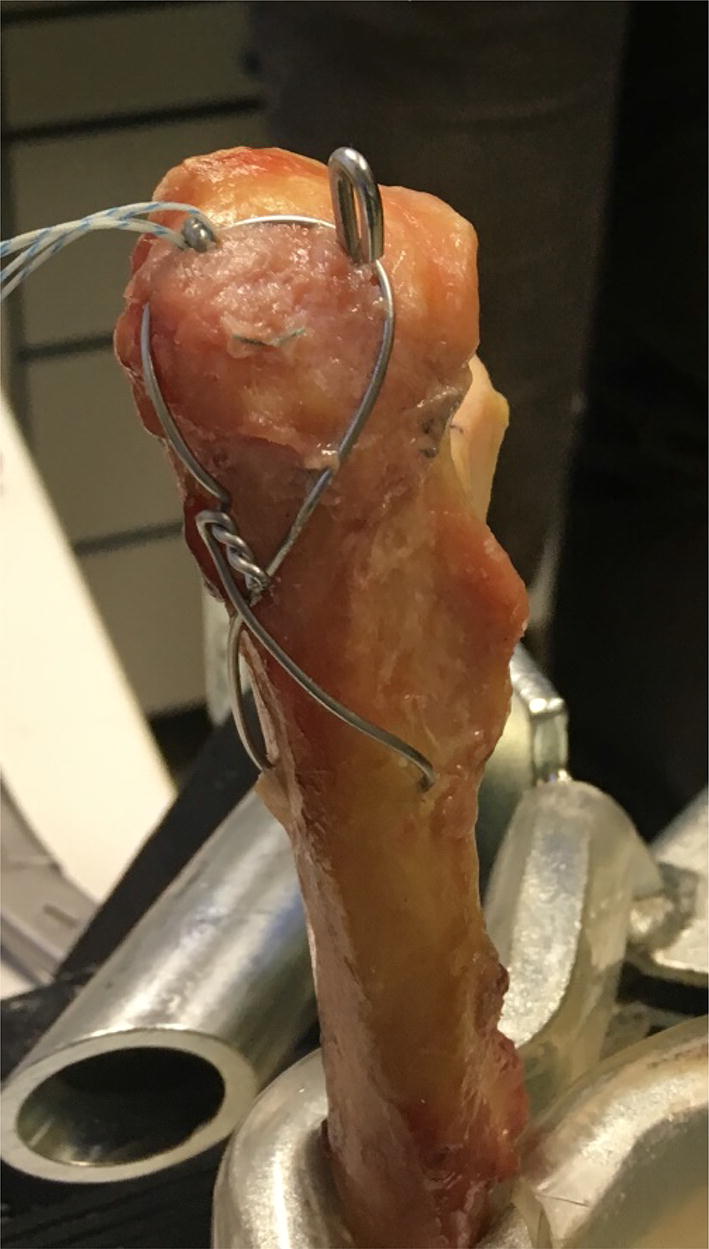
Test setup with the TBW of the proximal ulna with the bi- (radial, right) and tricortical (ulnar, left) k-wire

The ulnae were completely cleared of soft tissue by leaving only bone. The ulnae diaphysis was sawn 15 cm from the proximal end. Subsequently, the prepared specimens were implanted into bone cement (Dental Plaster Typ 4, Excalibur, Water/plaster ratio 22:100, Siladent, Dr. Böhme & Schöps GmbH, Germany) in an exact 90° position (Fig. 3). To reduce variation, the preparation was carried out by a single orthopedic surgeon in a standardized fashion. Todisco et al. had already proven that Hounsfield units (HU) measured in CT correlate highly with bone mineral density [11]. Therefore, the bone density of the specimens was measured by using a quantitative computed tomography (Somatom CT, Siemens, München, Germany, technical specifications: CTDI 4.53 vol*mGy, kV 80, mAs 180, .75 mm layer thickness). The bone density of all used proximal ulnas was on average 579 ± 186 (336–899) HU. Table 1 gives an overview.

**Table 1:** Overview of the donors with corresponding results and bone density

Donor	Side	Sex	Age	Maximum pullout strength—bicortical	Maximum pullout strength—tricortical	Bone density in HU
1 re	Right	Female	62	402.5	395.7	891
1 li	Left	Female	62	377.4	222.7	899
2 re	Right	Male	83	433.5	397.1	502
3 li	Left	Male	76	466.2	429.4	517
4 re	Right	Male	91	210.9	184.3	477
4 li	Left	Male	91	267.7	251.6	400
5 re	Right	Female	83	352.7	229.3	570
5 li	Left	Female	83	324.8	224.9	600
6 re	Right	Female	92	272.6	168.8	600
6 li	Left	Female	92	143.7	125.3	336

### Implants

For biomechanical testing, a 1.8-mm steel k-wire was used (Aesculap, Fa. Braun, Germany). The k-wires were implanted alternately for every fixation method in the radial and ulnar side randomly to avoid stability bias (advanced prepared lottery procedure). The wire was guided tangentially (about 20°), just below the joint surface until the wire perforated the second cortices of the ulna [7]. After that, the hole for the tension band (1.25 mm, Aesculap, Fa. Braun, Germany) was drilled tangential to the bone, passed through, twisted to create a figure of an 8, put over the proximal ends of the k-wires and closed with multiple twists. Later, the k-wires were oblique shortened 1.5 cm from ulna tip, bent 180° using a k-wire bending tool and inserted into the bone either bi- (without perforating the cortex of the ulna tip) or tricortical according to the group distribution (Figs. 1, 3).

### Test setup

**Fig. 4:**
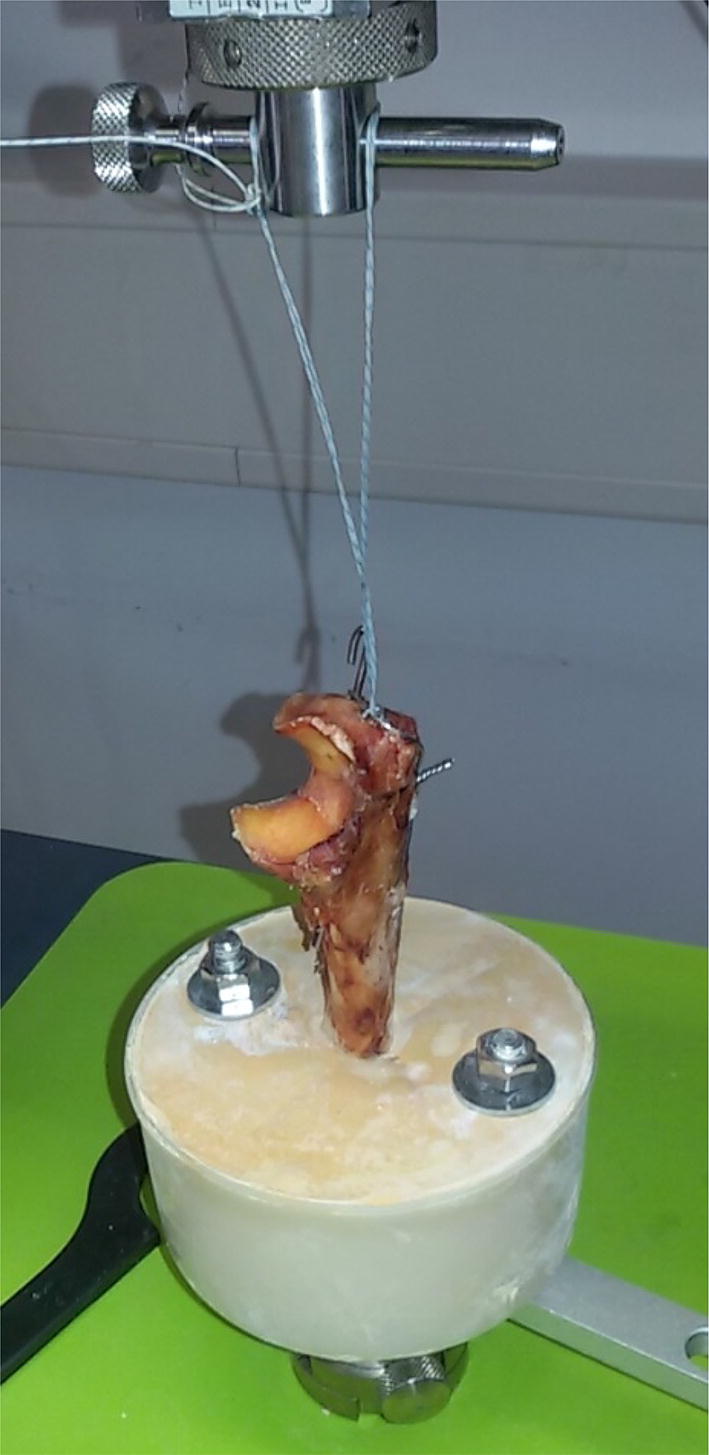
Test setup with the TBW of the proximal ulna under the uniaxial tension testing system (Zwick/Roell^®^) while fixing the tricortical k-wire with a highly cross-linked 2-mm FiberTape^®^

Uniaxial tension tests were performed using a Zwick/Roell^®^ series testing system (Z010, Zwick GmbH, Ulm, Germany) equipped with a 10-kN load cell. The prepared ulna bones were positioned under the tension arm and fixed with a steel screw/plate construction (Fig. 4). A highly cross-linked 2-mm FiberTape^®^ (Arthrex, München, Germany) was connected to the bi- or tricortical inserted k-wires and fixed to the tension arm of the Zwick^®^ testing system. The very flat and not bulky FiberTape^®^ was used to avoid any bias due to the minimal elevation of the wire in the bone. The tests were performed at 20 °C, 65% relative humidity. The k-wires were preloaded to 1 N and tested with a speed of 10 mm/min until failure.

Statistical analysis was performed with SPSS Statistics software (version 24; IBM, Armonk, NY, USA) for descriptive statistics. The significance level was chosen at *p* < .05 and all data are presented as mean with standard deviation (minimum−maximum). Univariate analysis of variance was carried out to compare the different k-wire fixations.

## Results

The average age of the used donors was 81.5 ± 11.5 (62–92) years. Three donors were females and three males. Ten k-wires were examined in BC and 10 in the TC group. Because each k-wire was used in every ulna, there was no bias in regard to age distribution and dominant handedness.

**Fig. 5:**
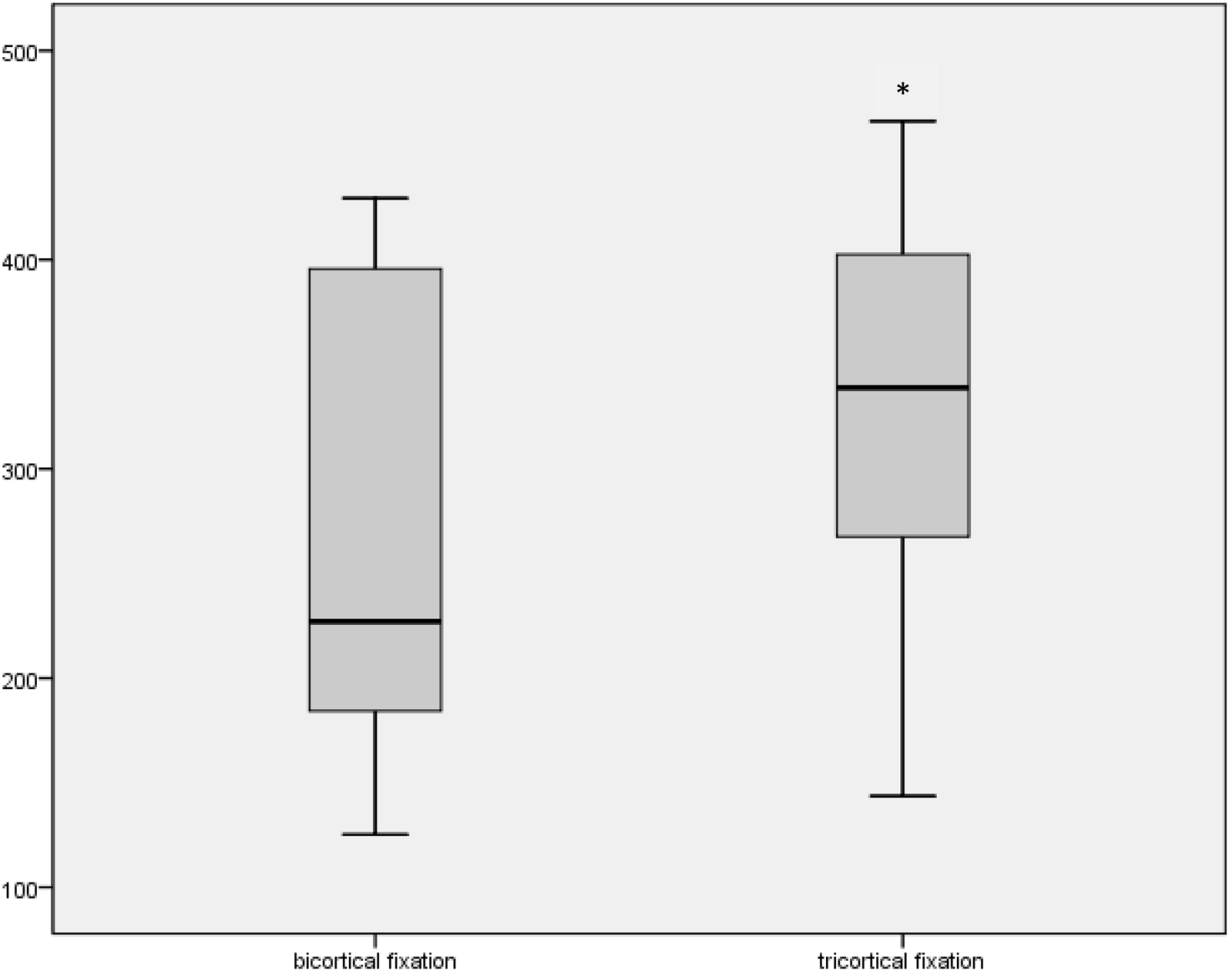
Boxplot of outcome parameter—maximum pullout strength in N: left bicortical fixation with 263 N (min: 125 N, max: 429 N, SD: 106 N) and right tricortical fixation with 325 N (min: 144 N, max: 466 N, SD: 102 N; *p* = .005)

All biomechanical tests of the k-wires were conducted successfully without tearing of the FiberTape^®^. The maximum pullout strength was 263 ± 106 (125–429) N in the BC and 325 ± 102 (144–466) N in the TC group (Fig. 5). Table 1 gives an overview. Using a paired *t* test, the tricortical group showed a significantly increased pullout strength (*p* = .005). There was no significant correlation between the measured bone density and the pullout strength (bicortical: *p* = .442 vs. tricortical: *p* = .124).

**Fig. 6:**
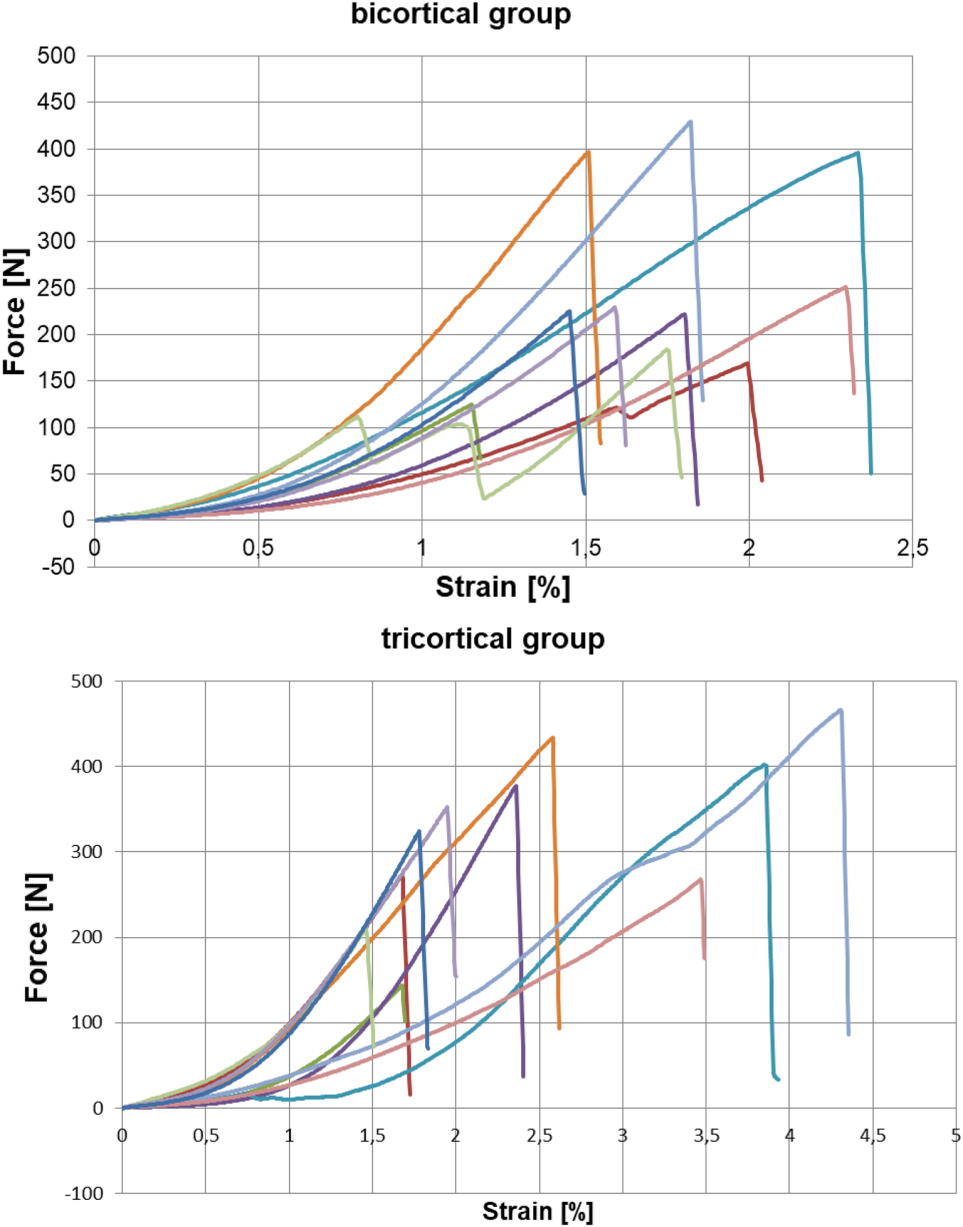
Force–strain curve **a** bicortical group, **b** tricortical group

The force–strain curves show a primary nonlinear and a secondary linear region. This can be explained by the elongation of the FiberTape^®^ at the beginning of the experiment. The start of a linear region in progress of the experiment indicates the transition to stiffness of the bone and finally the rapid decline with failure of the k-wire (Fig. 6).

## Discussion

Patients with a simple olecranon fracture are often treated with a TBW, since it is known as a biomechanically appropriate and cost-effective procedure [1]. Nevertheless, the technique is in detail more challenging than thought, resulting in a considerable high rate of implant-related complications like k-wire loosening and soft tissue irritation. It is therefore necessary to ensure that the inserted k-wires are securely fixed in the bone. It is known from clinical and radiographically studies that a transcortical fixation has a lower rate of pullout compared with intramedullary wires [8]. However, there is no study that evaluates different cortical fixation of k-wires in the bone. Therefore, we evaluated this for the first time in a biomechanical study and showed that a tricortical fixation of a k-wire in the bone has a significantly higher maximal pullout strength compared to a bicortical fixation.

The bone density of all used proximal ulnas was 579 HU, which is slightly less comparable biomechanical studies. Gruszka et al. evaluated in a comparison of TBW versus a novel olecranon tension plate (OTP) a mostly similar bone density of 694 HU for the OTP and 671 HU for the TBW group [2]. However, in the present study, the average age was 81.5 years higher than compared to Gruszka et al. [2].

In the literature, there is no comparative study of a biomechanical k-wire osteosynthesis. Saeed et al. evaluated, in a radiographical study, surgically modifiable factors related to spontaneous wire pullout in TBW [8]. They found in a multiple regression model in summary 7 variables affecting wire pullout. One of them was a higher pullout rate for medullary compared to transcortical wire positioning. However, medullary fixation means a monocortical fixation in the bone, which is obviously weaker than a transcortical fixation. Nevertheless, this supports the need for the present study which observed the differentiation between bi- or tricortical fixation. Macko et al. evaluated in a 5-year retrospective study of 20 olecranon fractures treated with primary open reduction using the AO technique of tension band wiring a prominence of the k-wires in 16 of 20 patients [4]. The authors describe in 12 of 16 cases already improper seating at the time of surgery, which underlines the need of stable fixation of the k-wires. In another retrospective study, Schneider et al. reviewed 239 TBW cases in patients with olecranon fractures or osteotomies in regard to operative imperfections [9]. The most frequent imperfection in 91% of all cases was the insufficient fixation of the proximal ends of the k-wires in 12 of 16. The conclusion of the authors was that TBW is not as easy as surgeons and the literature suggest.

Further, some other studies compare TBW with an olecranon tension plate (OTP). Gruszka et al. found no statistical significant loosening of fracture fragments in the articular surface comparing treatment with TBW and a novel low-profile OTP. Uhlmann et al. compared in a prospective study a percutaneous double-screw fixation (PDSF) versus TBW and determined a lower rate of implant removal in the PDSF Group and a significantly higher range of motion, although there was no significant difference in clinical scores [12].

The study has some limitations. We accepted an inaccurate anatomical direction of traction with respect to the muscle triceps. This was consciously chosen since not the tension band wiring itself but the biomechanical stability of a bi- versus tricortical fixation was evaluated. Furthermore, the fixation of the k-wires on the traverse with a FiberTape^®^ can be discussed. In our opinion, the small cross-sectional area of FiberTape^®^ avoids excessive lifting of the wire and thus the associated bias. Additionally, in the present study, only the pullout strength of the k-wires was investigated without an additional fracture placement. Although this does not reflect the clinical and surgical routine, it reduces the potential instability bias caused by the fracture and friction of the cerclage wire. A further limitation is that one specimen was used for every type of k-wire fixation (bi- and tricortical). However, bias was reduced by the random change of the wire between radial and ulnar for each specimen. Finally, it is not sure whether a significantly improved pullout strength in the biomechanical testing will result in better clinical performance of tricortical k-wire fixation.

## Conclusion

This study confirms for the first time biomechanical superiority of tricortical k-wire fixation in the olecranon when using a TBW and may justify the clinical use of this method.

## Data Availability

The material and the data are made available.
